# Commentary: Does Cognitive Behavior Therapy for psychosis (CBTp) show a sustainable effect on delusions? A meta-analysis

**DOI:** 10.3389/fpsyg.2016.00059

**Published:** 2016-02-01

**Authors:** Keith R. Laws

**Affiliations:** Psychology, University of HertfordshireHatfield, UK

**Keywords:** cbt, CBTp, delusions, follow-up, CBT for psychosis, schizophrenia, psychosis

In their meta-analysis, Mehl et al. (2015) examine whether CBT for psychosis is an effective intervention for reducing delusions. The authors reported a small but significant effect size for CBT in comparison to TAU at end-of-therapy (*k* = 13; *d* = 0.27) and at follow-up (*k* = 12; *d* = 0.25). By contrast, no significant benefit emerged for CBT when compared to active control conditions either at end-of-therapy (*k* = 8; *d* = 0.16) or at follow-up (*k* = 5; *d* = −0.04). The meta-analysis, however, contains errors and omissions that when rectified, cast doubt on the reliability of the reported significant effects comparing CBT to Treatment as Usual (TAU) at end-of treatment and at follow-up.

First, for the end-of-trial analysis, Mehl et al. demonstrate the presence of significant publication bias. Their funnel plot is asymmetric and a trim-and-fill analysis points to four possible missing unpublished studies (a large number when only 13 RCTs studies were analyzed). They note that when effect sizes for the four “missing” studies are included, the original effect size almost halves from *d* = 0.27 to *d* = 0.14. Mehl et al. did not, however, report the 95% Confidence Intervals and whether the revised effect size is significant (rather they report the standard error: *SE* = 0.12)—the required analysis reveals that the revised effect size of 0.14 becomes non-significant (95%CI −0.07 to 0.35).

Second, Mehl et al. also highlight the moderate level of heterogeneity in their end-of-trial analysis and attribute this to one outlying study by Kråkvik et al. (2013). After removing Kråkvik et al. they report that heterogeneity reduces with I2 falling from 42 to 11.7%. Key information concerning the revised effect size is, however, missing. A random effects recalculation shows that removal of Kråkvik reduces the effect size from 0.27 to 0.19 (95% CI 0.03 to 0.35), which is small but significant; however re-calculation of I2 did not seem to fall to 11.7% but 21.6%. Whatever the reduction in the percentage of I2, the key point here, is why Krakvik (*d* = 0.94) alone is considered an *outlier*, but not Foster (*d* = 0.90) or Waller et al. (*d* = 0.89). The removal of a single *outlier* here is somewhat opaque and atheoretical.

Third, Mehl et al. give only a fleeting mention to the most well-documented, “genuine” and significant source of heterogeneity in CBTp trials—whether outcomes are measured blind or not (see Lynch et al., 2010; Jauhar et al., 2014). Indeed, the issue of blinding pertains both to the issue of Kråkvik et al. and the asymmetric funnel plot mentioned above. Mehl et al. state that “Only one of these studies did not use single-blind assessment (Foster et al., 2010)”; however, it is clear that Mehl and colleagues misclassify two non-blind trials as blind. In their discussion, Mehl et al. note that Kråkvik et al. (2013) “produced a quite large effect size (*d* = 0.94), which might also have been influenced by difficulties in maintaining the blinding procedure.” If we turn to the Kråkvik et al paper itself, those authors clearly state that “All four professionals were trained in the use of assessment measures, but *it was not possible to keep them blind to the treatment condition*.” Mehl et al. also incorrectly classify Waller et al. (2015) as a blind trial when it was non-blind. These 3 of 13 non-blind trials (Foster 0.90; Kråkvik 0.94; Waller 0.89) produced the largest effects reported for CBT on delusions. Further analysis of Mehl et al.'s data shows that blind trials (*k* = 9) elicit a non-significant effect size of *d* = 0.13 (95% CI −0.028 to 0.29) while non-blind trials (*K* = 4 adding Lincoln, which is self-rating) produce an effect size five-times larger with *d* = 0.65 (95% CI 0.21 to 1.09). Additionally, it is notable that amongst the nine blind trials, heterogeneity is virtually non-existent I2 = 4.8. In sum, the minority of non-blind trials underpins the inflation of effect sizes and their reported heterogeneity.

Turning to the follow-up analysis, where Mehl et al. claim an overall significant effect of CBT on delusions with *d* = 0.25. Their analysis reports a significant effect size of 0.43 for Turkington et al. (2006) which is the largest sample included and hence most heavily weighted effect size in their meta-analysis. This effect size is, however, difficult to reconcile with the data presented in the Turkington paper itself- where the Psychotic Symptom Rating Scale (PSYRATS: Haddock et al., 1999) delusion score was not only non-significant, but in the opposite direction—with a greater decrease of delusions in controls than CBT (confirmed in personal communication by one of the authors). A recalculation using the Turkington data reveals an effect size of *d* = −0.11 (−0.33 to 0.11) in favor of controls. If we add this new value to replace the apparently erroneous effect size reported by Mehl et al. a random effects model now shows the overall effect size reduces to 0.16 (−0.03 to 0.34) and becomes non-significant.

To summarize, examination of Mehl et al.'s end-of-trial data comparing CBT and TAU shows that if the overall effect size is adjusted for potential publication bias, then it becomes non-significant. Further analysis of the same data also shows that the significant heterogeneity reported by Mehl et al. is likely to reflect the inclusion of 4 non-blind trials, which elicit effects sizes five-times larger than in blinded trials. Analysis of nine blind trials revealed no heterogeneity and no CBT efficacy. Turning to the follow-up data, adjusting the effect size for Turkington et al. (2006) means the overall CBT efficacy becomes non-significant. In other words, CBT fails to reduce delusional thinking compared to “active” controls at either end-of-trial or at follow-up, and further fails to reduce delusional thinking when compared to TAU at follow-up and shows no efficacy at end-of-trial except where there is high risk of bias.

## Author contributions

The author confirms being the sole contributor of this work and approved it for publication.

### Conflict of interest statement

The author declares that the research was conducted in the absence of any commercial or financial relationships that could be construed as a potential conflict of interest. The reviewer, Maria Semkovska, and handling Editor declared their shared affiliation, and the handling Editor states that the process nevertheless met the standards of a fair and objective review.
